# Mitochondrial Respiratory Function Induces Endogenous Hypoxia

**DOI:** 10.1371/journal.pone.0088911

**Published:** 2014-02-21

**Authors:** Sara Prior, Ara Kim, Toshitada Yoshihara, Seiji Tobita, Toshiyuki Takeuchi, Masahiro Higuchi

**Affiliations:** 1 Department of Biochemistry and Molecular Biology, University of Arkansas for Medical Sciences, Little Rock, Arkansas, United States of America; 2 Department of Chemistry and Chemical Biology, Graduate School of Engineering, University of Gunma, Kiryu, Gunma, Japan; 3 Department of Molecular Medicine, Institute for Molecular and Cellular Regulation, University of Gunma, Maebashi, Gunma, Japan; Duke University Medical Center, United States of America

## Abstract

Hypoxia influences many key biological functions. In cancer, it is generally believed that hypoxic condition is generated deep inside the tumor because of the lack of oxygen supply. However, consumption of oxygen by cancer should be one of the key means of regulating oxygen concentration to induce hypoxia but has not been well studied. Here, we provide direct evidence of the mitochondrial role in the induction of intracellular hypoxia. We used Acetylacetonatobis [2-(2′-benzothienyl) pyridinato-k*N*, kC3’] iridium (III) (BTP), a novel oxygen sensor, to detect intracellular hypoxia in living cells via microscopy. The well-differentiated cancer cell lines, LNCaP and MCF-7, showed intracellular hypoxia without exogenous hypoxia in an open environment. This may be caused by high oxygen consumption, low oxygen diffusion in water, and low oxygen incorporation to the cells. In contrast, the poorly-differentiated cancer cell lines: PC-3 and MDAMB231 exhibited intracellular normoxia by low oxygen consumption. The specific complex I inhibitor, rotenone, and the reduction of mitochondrial DNA (mtDNA) content reduced intracellular hypoxia, indicating that intracellular oxygen concentration is regulated by the consumption of oxygen by mitochondria. HIF-1α was activated in endogenously hypoxic LNCaP and the activation was dependent on mitochondrial respiratory function. Intracellular hypoxic status is regulated by glucose by parabolic dose response. The low concentration of glucose (0.045 mg/ml) induced strongest intracellular hypoxia possibly because of the Crabtree effect. Addition of FCS to the media induced intracellular hypoxia in LNCaP, and this effect was partially mimicked by an androgen analog, R1881, and inhibited by the anti-androgen, flutamide. These results indicate that mitochondrial respiratory function determines intracellular hypoxic status and may regulate oxygen-dependent biological functions.

## Introduction

Oxygen concentration within the cell regulates many key biological functions including HIF-1α activation, glucose transport [1], potassium pump activity, intracellular calcium concentration [2], P450 family enzymes [3], and HMGR (3-hydroxy-3-methyl-glutaryl-CoA reductase) expression [4], [5]. In cancer, oxygen concentration deep inside the lesion is low and can elicit a hypoxic response in cells, however, other mechanisms are involved in the regulation intracellular hypoxic status [6]. Mitochondrial DNA (mtDNA) encodes thirteen proteins that form essential subunits of the mitochondrial respiratory chain complexes along with the subunits encoded by the nuclear DNA. We previously showed that in prostate cancer cells from patients, reduction in mtDNA content is associated with cancer progression as estimated by Gleason grade [4]. A report by Lu *et al.* showed that reduction of mtDNA content induced the Warburg effect (a reduction in oxidative phosphorylation with an increase in fermentative glycolysis) [7]. We also reported that reduction of mtDNA content induces an anti-apoptotic phenotype [8], [9], cancer progression phenotypes [10]–[12], and cancer progression signals such as NF-κB [13], AP-1 [13], ERK [11], JNK [11], and AKT [8]. We have also recently reported on the ability oxygen to regulate the degradation of HMGR leading to the activation of Ras in prostate cancer cells [4]. Additionally, Nguyen and colleagues have reported the hypoxia stimulated degradation of HMGR [5]. These findings led us to hypothesize that the mitochondria play a central role in cancer progression through the regulation of intracellular oxygen concentration. To this end, we employed the Oxoplate system (described in detail in the methods and in Cook *et al.* [4]) to evaluate the ability of cells to change the oxygen concentration in the media surrounding the cells in an open system. To evaluate the intracellular hypoxic status in living cells via microscopy we utilized BTP, which localizes to the ER (described in detail below and in Zhang *et al.* [14]). Pimonidazole has been utilized to detect cellular hypoxia, but we elected not to use it in this study since pimonidazole detects protein adducts induced by hypoxia rather than being a direct detector of cellular oxygen [15]–[17]. In our studies we elected to use BTP in order to observe changes in intracellular hypoxia in real time in living cells. Our data provide the first direct evidence that mitochondrial function can regulate intracellular hypoxic status and that this ability can be controlled by glucose availability and androgen in prostate cancer.

## Materials and Methods

### Materials

Uridine, rotenone, glucose solution, pyruvate, hydroxyurea, flutamide, CoCl_2_, R1881, and sodium sulfite were purchased from Sigma-Aldrich.

### Cell Culture

MCF-7, MDAMB231, and PC-3 were purchased from ATCC. LNCaP and C4-2 were purchased from UROCOR. MDAMB231 and LNCyb were cultured in Dulbecco’s modified Eagle medium (DMEM) with glucose (4.5 g/l), sodium pyruvate (110 mg/l) and GlutaMAX (Life Technologies) plus 5% heat-inactivated fetal calf serum (FCS) (Life Technologies). MCF-7 cells were cultured in the same conditions as listed above but also in the presence of 0.01 mg/ml recombinant human insulin (Life Technologies). LNCaP, PC-3, and C4-2 were maintained in RPMI (Life Technologies) plus 5% FCS. LNρ0-8 cells were cultured in DMEM with glucose (4.5 g/l), sodium pyruvate (110 mg/l), and GlutaMAX plus 10% FCS and supplemented with uridine (50 µg/ml). All cell lines were maintained at 37°C with room air plus 5% CO_2_ unless noted for specific experiments.

### Determination of Oxygen Concentration Surrounding Cells Using Oxoplate-Oxoplate

OP96F plates were purchased from PreSens. The Oxoplate system has been described in detail by Cook *et al.* [4]. Briefly, wells containing 200 µl of air-saturated (K_100_) and oxygen-free water (K_0_) served as standards for high- and low-oxygen conditions, respectively. Oxygen-free water was prepared by the addition of sodium sulfite (1%); the well was then overlaid with mineral oil (50 µl) to prevent diffusion of oxygen into the water during the course of the experiment. The remaining wells were open to the environment inside the plate reader so that atmospheric oxygen was able to diffuse into the media of the sample wells. Cells were grown in 10 cm culture dishes under normal culture conditions until the cells had reached approximately 80% confluence and then collected by trypsinization. Cells were added to the Oxoplate at a concentration of 1×10^6^ cells/ml in RPMI plus 5% FCS (200 µl final volume). Fluorescence in each well was then measured every 5 minutes for 3 hours at 37°C in a plate reader (Synergy HT, BIO-TEK) in dual kinetic mode. The oxygen concentration in the wells at each time point was calculated, in micromoles, using the following equation:
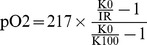



K_100_ is the I_R_ of the well containing air-saturated water. K_0_ is the I_R_ of the well filled with oxygen-free water. I_R_ for each sample is calculated by dividing the fluorescence of the indicator dye (544/645 nm) by the fluorescence of the reference dye (544/590 nm). All samples were run in triplicate.

### Determination of Oxygen Consumption Rate using Oxytherm

Oxygen consumption rate in a closed environment was measured using the Oxytherm system (Hansatech). Samples were run at a cell concentration of 1×10^6^ cells/ml for 10 minutes at 37°C in the appropriate culture media for each cell line. All samples were run in triplicate. Further detailed methods were described in Cook *et al.* [4].

### Laser Confocal Microscopy

Unless otherwise noted, cells were maintained in a stage-mounted atmospheric box (Pathology Devices) at 37°C, 5% CO_2_, and 75% humidity during the course of the experiments. All samples were analyzed on an Olympus Fluoview FV1000 laser confocal microscope. Acetylacetonatobis [2-(2′-benzothienyl) pyridinato-k*N*, kC3’] iridium (III) (BTP) (515 excitation/620 emission) was utilized in all confocal experiments at a concentration of 5 µM. BTP has been described in detail by Zhang *et al.* [14]. BTP is a phosphorescent compound that is phosphorescent in low-oxygen conditions and is quenched in the presence of oxygen. The extent of quenching is dependent upon intracellular oxygen concentration. Samples were incubated in the presence of BTP for 1 hour before imaging (under normoxic (20% O_2_), hypoxic (0.2% O_2_), or hyperoxic (40% O_2_) conditions as detailed for specific experiments). In all experiments, cells were plated at a cell density of 1×10^5^ cells in 3 cm dishes with inset cover slips (Mattek). LNCaP and MCF-7 cells were incubated for at least for 48 hours, depending on cell adherence and morphology, before the beginning of experiments. All other cell lines were plated 24 hours before the beginning of experiments. At the start of each experiment samples were incubated with BTP plus any additional experimental conditions detailed below for one hour before viewing. Experiments that were carried out in the absence of serum or in the presence of R1881 or flutamide were incubated for twenty-four hours before the addition of BTP. Images from all microscopy experiments were processed using the FV10-ASW 3.1 Viewer (Olympus). Cell phosphorescence for 10 cells per plate was quantified using ImageJ. Cell phosphorescence was calculated as corrected total cellular phosphorescence (CTCP) according to the following equation:
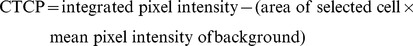



This method takes into account the area of the cell when determining pixel intensity of a cell. Images have been false-colored green for easier viewing.

### Detection of HIF-1α by Western Blotting

For a positive control for the detection of HIF-1α, cells were treated with 200 µM CoCl_2_ for 6 hours before nuclear extract collection. For samples that were exposed to hypoxia, the cells were incubated at 0.2% oxygen (5% CO_2_, 94.8% N_2_) for 6 hours before nuclear extract collection. Nuclear extracts were collected by first addition of 10 mM HEPES buffer plus 1 mM DTT and protease and phosphatase inhibitors (100X Halt protease and phosphate inhibitor cocktail, #1861281 Thermo Scientific) at a ratio of 400 µl per 2×10^6^ cells. After the cells were allowed to swell for 15 minutes 10% NP-40 was added at a ratio of 25 µl per 2×10^6^ cells. After centrifugation the cytosolic fraction was removed and the pellet was again resuspended in 10 mM HEPES and centrifuged again to ensure better removal of cytosolic components. The nuclear pellet was then resuspended in 20 mM HEPES buffer plus DTT and protease and phosphatase inhibitors at a ratio of 25 µl per 2×10^6^ cells for 15 minutes. Following centrifugation the nuclear fraction was collected. 20 µg of protein from each sample was then separated on an 8% SDS-PAGE gel. Samples were then electro-blotted to a PVDF membrane. The membrane was blocked with 5% milk in TBST. The blot was probed with anti-HIF-1α antibody (Santa Cruz Biotechonolgy) (1∶250 dilution in blocking buffer). The secondary antibody was HRP-linked anti-mouse (2∶5,000). PCNA (Santa Cruz Biotechnology)served as a loading control (1∶200 dilution). Bands were then visualized using ECL Prime (GE Healthcare) and the ImagQuant LAS 4000 phosphoimager (GE Healthcare). Densitometric analysis was carried out using ImageJ. Band intensities were normalized to the corresponding PCNA loading control intensities.

### BTP Incorporation

LNCaP and LNρ0-8 cells were seeded on a 96-well plate at a cell density of 1×10^6^ cells in 200 µl. The cells were then allowed to attach overnight, and the next day the media was removed and the cells were recovered with PBS containing 100 nM BTP. After one hour incubation the supernatant was removed and transferred to a new 96-well plate. The absorbance was then measured at 483 nm. Incorporation of BTP into the cells was determined as the difference in the OD of PBS that had been added to the cells versus cell-free control.

### BTP Synthesis

BTP was synthesized according to the literature [18]. A mixture of 2-(benzo[b]thiophen-2-yl)pyridine (460 mg, 2.2 mM), IrCl_3_•3H_2_O (390 mg, 1 mM), 2-ethoxyethanol (30 ml), and distilled water (10 ml) was heated at reflux for 15 h. After cooling, the precipitate formed was filtered to give a chloro-bridged dimer and washed thoroughly with methanol and n-hexane. To the mixture of the chloro-bridged dimer (260 mg, 0.2 mM) and Na_2_CO_3_ (170 mg, 1.6 mM) were added 2-methoxyethanol (30 ml) and acetyl acetone (1.0 ml, 9.5 mM), and then the slurry was refluxed for 2 h. After the mixture was cooled, the solvent was evaporated to dryness under reduced pressure. The crude product was purified by silica-gel column chromatography using chloroform as eluent. The product was obtained as a brown powder (180 mg, 0.25 mM, 63%), and was identified by 1H NMR spectroscopy. 1H NMR (300 MHz, CDCl3) δ: 8.43 (d, 2H, J = 5.4 Hz), 7.77 (t, 2H, J = 8.4 Hz), 7.63 (d, 4H, J = 7.2 Hz), 7.07–6.99 (m, 4H), 6.80 (t, 2H, J = 7.8), 6.20 (d, 2H, J = 8.1 Hz), 5.26 (s, 1H), 1.78 (s, 6H).

### Statistical Analysis

Statistical significance was calculated using the two-tailed Student’s *t*-test. *P*-values below 0.05 were considered to be statistically significant.

## Results and Discussion

### Association of Oxygen Consumption Rate and the Induction of Hypoxia Inside of and Surrounding the Cells without Exogenous Hypoxia

**Figure 1:**
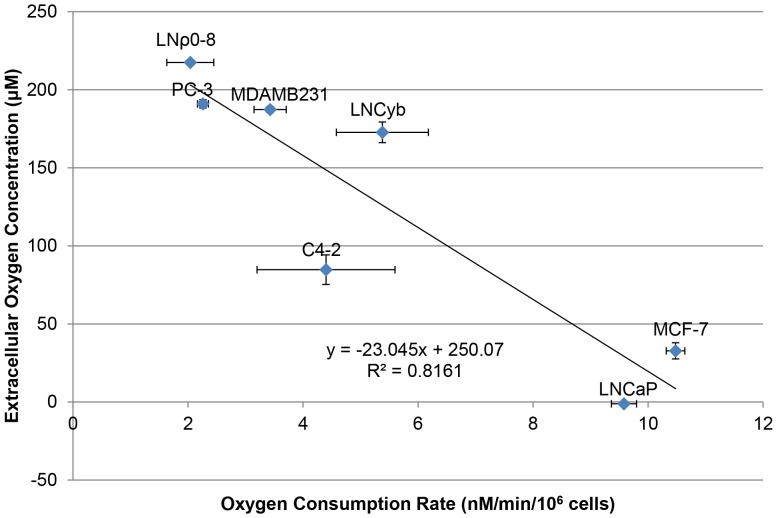
Oxygen concentration surrounding the cells and oxygen consumption rates in various cell lines. Correlation between oxygen consumption rate and oxygen concentration surrounding the cells after 3 = −23.045x +250.07 with a R^2^ value of 0.8161. Error bars represent standard error.

Table 1 shows all cell lines used in this study in conjunction with a characteristics and origins of cell lines based on a search of the literature. Our previous report suggests that mtDNA content is associated with oxygen consumption rate [4]. We hypothesized that consumption of a large amount of oxygen in the cells with high mtDNA content induces hypoxia. To investigate this hypothesis, we measured oxygen consumption rate using Oxytherm (closed system) and oxygen concentration surrounding the cells in a 96-well plate using Oxoplate in an open environment. In the Oxoplate system oxygen is able to diffuse into the media as shown with the diffusion of oxygen into wells containing water with 2.5 mg/ml of sodium sulfite (Figure S1). Figure 1 shows the correlation between oxygen concentration surrounding the cells and oxygen consumption rate. We found that the prostate cancer cell line, LNCaP [19] with high mtDNA content (in comparison with normal prostate tissue) [4], [20], showed strong hypoxia surrounding the cells (although, surface of the culture medium is normoxic) and a high rate of oxygen consumption (Fig. 1). The prostate cancer cell line, PC-3 [21] with low mtDNA content [4] had a limited ability to consume oxygen and induce hypoxia in the media surrounding the cells as compared with LNCaP (Fig. 1). C4-2 cells (prostate cancer cell line) [22]with a moderate level of mtDNA content (as compared to LNCaP (high) and LNρ0-8 (low)) [4] showed a moderate extracellular hypoxia-inducing ability with a moderate rate of oxygen consumption (Fig. 1) as compared with LNCaP. The breast cancer cell line, MCF-7 [23], had a strong extracellular hypoxia-inducing ability but was not as strong as LNCaP (Fig. 1). The estrogen receptor-negative breast cancer cell line, MDAMB231 [24], [25], had a limited ability to induce hypoxia surrounding cells and to consume oxygen as compared with MCF-7 (Fig. 1). Oxygen concentration surrounding the cells in the Oxoplate was well correlated with oxygen consumption rate (Figure 1, R^2^ = 0.82). Induction of hypoxia surrounding cells is likely to be caused by the high oxygen consumption of cells combined with low oxygen diffusion [26].

**Table 1:** Characteristics of cancer cell lines used.

Cell Line	Phenotype	Origin
LNCaP (prostate)	Well-differentiated	Lymph node metastasis [19]
LNρ0-8 (prostate)	Poorly-differentiated	mtDNA depleted LNCaP [27]
LNcyb (prostate)	Moderately-differentiated	mtDNA replete LNρ0-8 [27]
C4-2 (prostate)	Moderately-differentiated	Castration resistant LNCaP from mouse bone [22]
PC-3 (prostate)	Poorly-differentiated	Bone metastasis [21]
MCF-7 (breast)	Well-differentiated	Pleural effusion [23]
MDAMB231 (breast)	Poorly-differentiated	Pleural effusion [24], [25]

**Figure 2:**
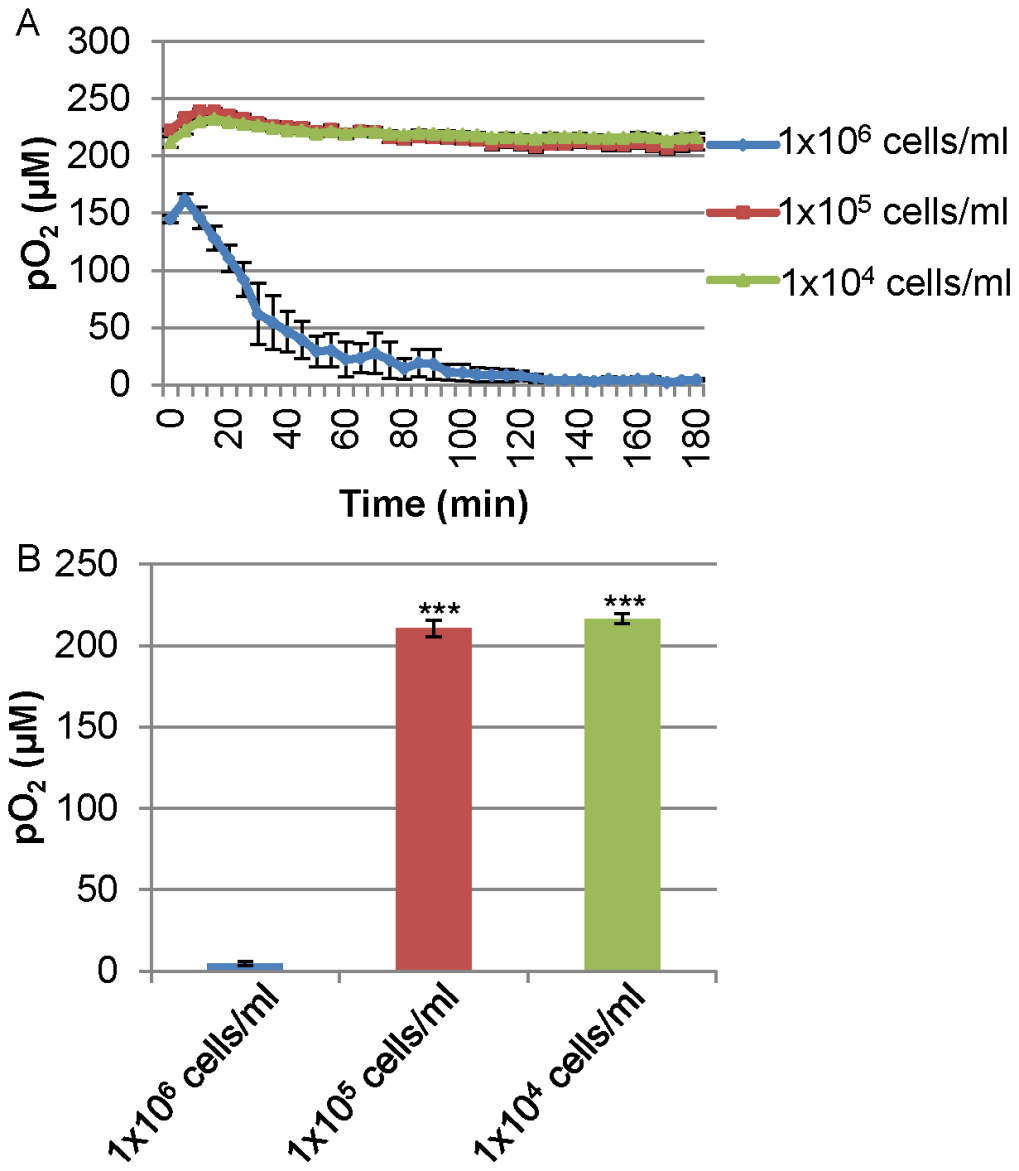
Oxygen concentrations surrounding the cells with different cell densities. (A) Time dependent changes in oxygen concentrations surrounding cells at an indicated cell density of LNCaP. (B) Oxygen concentration at 180 minutes from panel A (*n* = 3, ***P<0.001 when compared to 1×10^6^ cells/ml). Error bars represent standard error.

**Figure 3:**
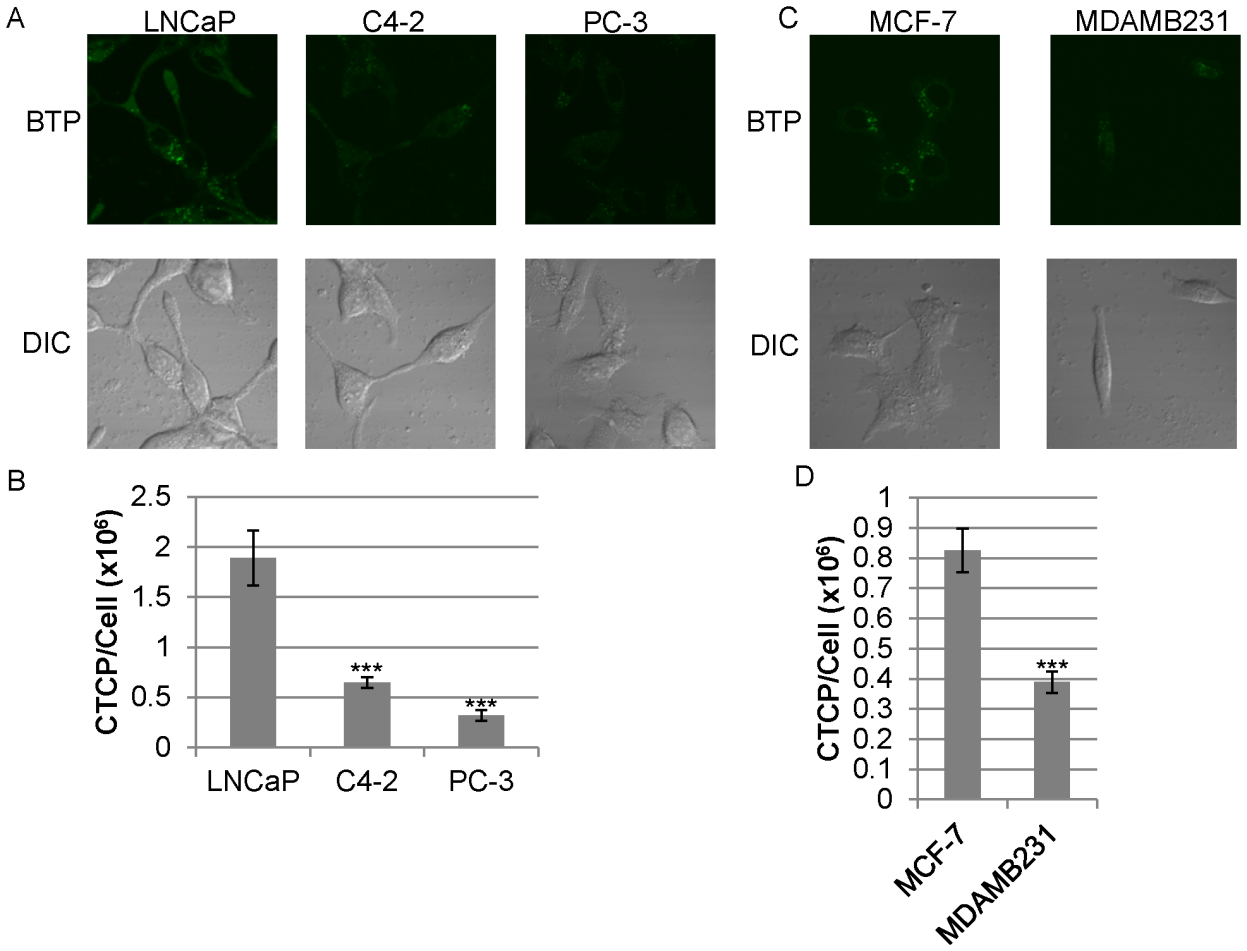
Intracellular oxygen status of various cell lines. (A) Detection of intracellular hypoxia by BTP in prostate cancer cell lines LNCaP, C4-2 and PC-3 (upper panels). DIC images show the positions of imaged cells (lower panels). (B) Quantification of BTP phosphorescence in panel A (*n = *10, ***P<0.001 when compared to LNCaP). (C) Detection of intracellular hypoxia by BTP in MCF-7 and MDAMB231 (upper panels). DIC images show the positions of imaged cells (lower panels). (D) Quantification of BTP phosphorescence in panel C (*n* = 10, **P<0.01 when compared to MCF-7). Error bars represent standard error.

We observed that the ability of LNCaP to induce hypoxia surrounding cells is cell number dependent. At low cell numbers (1×10^5^ and 1×10^4^ cells/ml) LNCaP was unable to induce hypoxia surrounding cells in the Oxoplate system (Fig. 2A–B). We then investigated whether cells with high oxygen consumption could be hypoxic intracellularly even though extracellular oxygen concentration surrounding cells is high in the low cell density. To examine intracellular hypoxic status, we employed the phosphorescent, hypoxia-sensing dye, BTP (described in detail in methods). This dye is phosphorescent in the absence of oxygen, and it is quenched by oxygen. Thus, more intense phosphorescence indicates a lower intracellular oxygen concentration [14]. Fig. 3A shows micrographs of cells stained with BTP (upper panels) plus DIC images (lower panels) showing the position of the cells. Fig. 3B is a quantification of BTP phosphorescence from panel A (described in detail in the methods) reported as average corrected total cellular phosphorescence (CTCP) ± the standard error. Quantification of BTP phosphorescence was carried out to allow for easier interpretation of the data. LNCaP exhibited high BTP intensity indicating stronger hypoxia under exogenously normoxic conditions than C4-2 and PC-3, (Fig. 3A–B, P<0.001, *n* = 10). MCF-7 was significantly more hypoxic than MDAMB231 (Fig. 3C–D, P<0.01, *n* = 10). As demonstrated above in Fig. 3A–B, LNCaP was able to induce intracellular hypoxia despite the normoxic condition surrounding the cells (Fig. 2A–B, Fig. 3A–B). This intracellular hypoxia in LNCaP and MCF-7 at low cell density is likely to be caused by the high oxygen consumption of mitochondria as compared with oxygen incorporation through the membrane. To investigate whether BTP is incorporated in similar amounts in different cells, we used LNCaP, which shows the highest BTP staining, and LNρ0-8, which shows lowest. To 1×10^6^ cells in 0.2 ml, we added 100 nM BTP. Similar amounts of BTP was incorporated to LNCaP and LNρ0-8 (23.6 (average) ±0.6 (SE) % and 24.9 (average) ±2.2 (SE) %), respectively. In light of these results, we decided to delve further into the mechanisms of how intracellular hypoxia is regulated.

### Mitochondrial Respiratory Function Induces Intracellular Hypoxia

**Figure 4:**
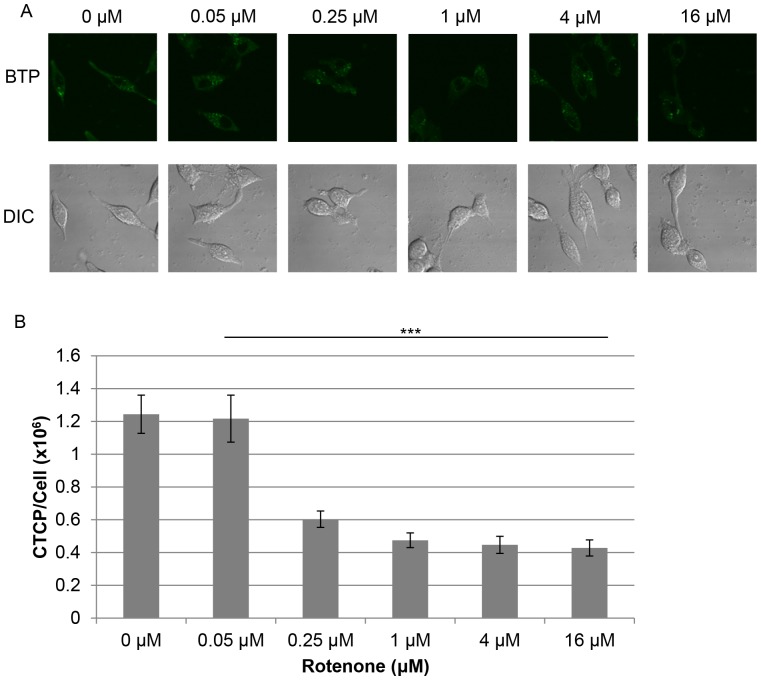
Intracellular hypoxia was dependent on mitochondrial respiration. (A) BTP phosphorescence of cells treated with indicated concentrations of rotenone. (B) Quantification of results in panel A (*n* = 10, ***P<0.001 when compared to 0 nM control). Error bars represent standard error.

**Figure 5:**
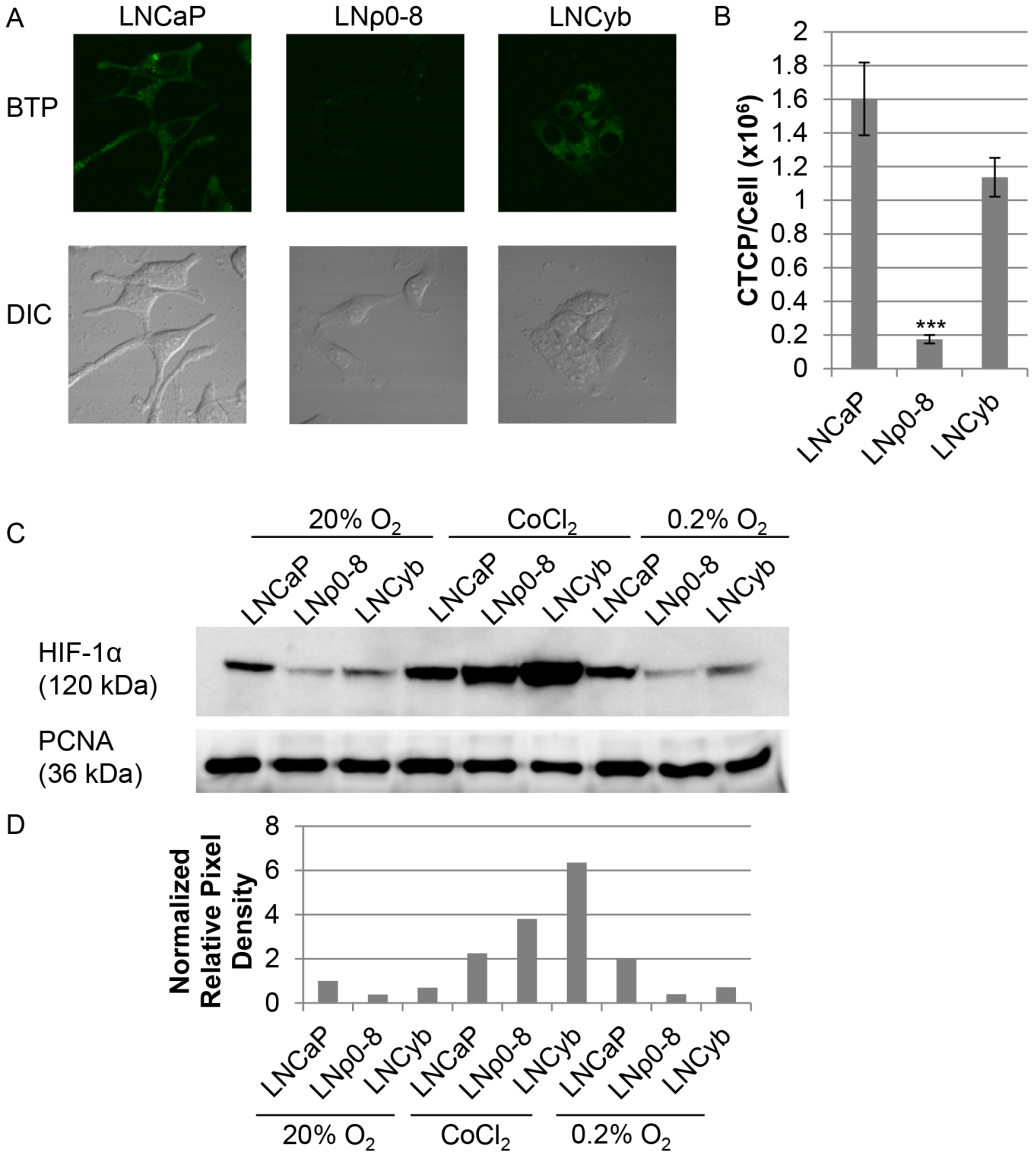
Reduction of mtDNA content reversibly inhibited intracellular hypoxia. (A) BTP phosphorescence of LNCaP, LNρ0-8, and LNCyb under normoxic conditions. (B) Quantification of BTP phosphorescence in panel A (*n = *10, ***P<0.001 when compared to LNCaP). Error bars represent standard error. (C) Western blot showing HIF-1α expression in nuclear extracts from LNCaP, LNρ0-8, and LNCyb cells under normal incubation conditions (left). Nuclear extracts from all three cells lines following treatment with CoCl_2_ served as positive controls for the detection of HIF-1α (center). All three cell lines were also exposed to hypoxia (0.2% O_2_) for 6 hours (right). PCNA served as a loading control. (D) Densitometric analysis of western blotting results in C. Band intensities were normalized to corresponding PCNA loading control band.

To demonstrate that mitochondrial respiratory function is the key mediator in the regulation of intracellular hypoxia, we used rotenone (a specific inhibitor of mitochondrial respiratory chain complex I) to inhibit respiratory function in LNCaP cells. Following treatment with rotenone, we observed a marked decrease in BTP phosphorescence relative to controls (Fig. 4A–B, P<0.001, *n* = 10) indicating that rotenone inhibited oxygen consumption and decreased the level of intracellular hypoxia in a dose-dependent manner. Additionally, we investigated how changes in the mtDNA content of the cells can influence intracellular hypoxia. Mitochondrial respiratory function is eliminated in the mtDNA deficient LNρ0-8 (derived from LNCaP) cell line because of the loss of the 13 mitochondrial respiratory proteins encoded in mtDNA. While lacking mtDNA, these cells still possess mitochondrial structures [4]. Respiratory activity is recovered, at least partially, in mtDNA reconstituted LNCyb cell line [4], [27]. LNCaP exhibited strong intracellular hypoxia under exogenous normoxic conditions (Fig. 5A–B). LNρ0-8 cells exhibited minimal intracellular hypoxia above background levels and induction of hypoxia is greatly reduced as compared with LNCaP (Fig. 5A–B, P<0.001, *n = *10). LNCyb exhibited intracellular hypoxia, but this hypoxia was not quite as strong as in LNCaP (Fig. 5A–B). These results indicate that intracellular hypoxia was dependent on mitochondrial respiratory function which is regulated by mtDNA content. The intermediate level of intracellular hypoxia observed in LNCyb was expected, based on previous work with LNCyb cells in our lab; these cells possess a phenotype that is between LNCaP and LNρ0-8 in terms of oxygen consumption [4]. Taken together, these data indicate that mitochondrial respiratory function is required to induce intracellular hypoxia.

### HIF-1α Activation in LNCaP Cells is Mitochondrial Respiration Dependent

HIF-1α is considered to be one of the primary regulators of the cell’s response to low oxygen tension [28]. HIF-1α stability is tightly linked to cellular oxygen availability via its regulation by oxygen-dependent enzymes and is stabilized under exogenous hypoxic conditions [29]. Stable HIF-1α is able to translocate into the nucleus where it serves as transcription factor for many hypoxia-regulated genes. Mechanisms of non-hypoxic regulation have also been documented [28]. Regulation of HIF-1α by mitochondrial function has also been implicated by others [29]. Therefore, we investigated whether the intracellular hypoxia that we observed in LNCaP cells induces HIF-1α stabilization under normal culture conditions. To evaluate HIF-1α activation, we examined the expression of HIF-1α in the nuclei of LNCaP, LNρ0-8, and LNCyb by western blotting. All three cell lines treated for 6 hours with CoCl_2_ served as a positive control for the detection of HIF-1α (Fig. 5C–D) as this compound inhibits the degradation of endogenously produced HIF-1α. As expected, intracellularly hypoxic LNCaP (Fig. 5C–D) showed HIF-1α expression in the nucleus under normal culture conditions (Fig. 5C–D). LNρ0-8, which is normoxic intracellularly (Fig. 5C–D), showed only a slight amount of detectable HIF-1α in comparison to LNCaP. LNCyb, with an intermediate level of intracellular hypoxia, showed a slight increase in HIF-1α expression relative to LNρ0-8. The low, but still detectable, levels of HIF-1α found in LNρ0-8 may be the result of remaining oxygen consumption without mtDNA-encoded mitochondrial respiratory chain proteins or an underlying non-hypoxic regulation [29]. However, the majority of the HIF-1α level in the nucleus appears to be regulated by mitochondrial function (and therefore intracellular hypoxia) as evidenced by the partial restoration of HIF-1α in LNCyb. In the CoCl_2_ treated controls LNCaP showed only a slight increase in HIF-1α expression (Fig. 5C–D). This suggests that HIF-1α levels may already be near the maximum in LNCaP cells under normal incubation conditions. As expected, CoCl_2_ stabilized HIF-1α in LNρ0-8 showing that these cells endogenously produce HIF-1α but it is degraded in the absence of COCl_2_ (Fig. 5C–D). HIF-1α expression was also increased in LNCyb cells, as expected, upon treatment with CoCl_2_ (Fig. 5C–D). Additionally, HIF-1α expression was enhanced in LNCaP and slightly enhanced in LNCyb upon exposure to 0.2% oxygen for six hours (Fig. 5C–D). LNρ0-8 cells showed only a very slight increase in HIF-1α following exposure to hypoxia (Fig. 5C–D). This is in agreement with previous findings by others showing that HIF-1α cannot be strongly induced under hypoxia in cells lacking mitochondrial function [30]. These results suggest that intracellular hypoxia determined by mitochondrial respiratory function regulates HIF-1α activation leading to hypoxia-related gene expression.

### Mitochondrial Function is Still Required to Induce Strong Intracellular Hypoxia, even under Exogenous Hypoxic Condition

**Figure 6:**
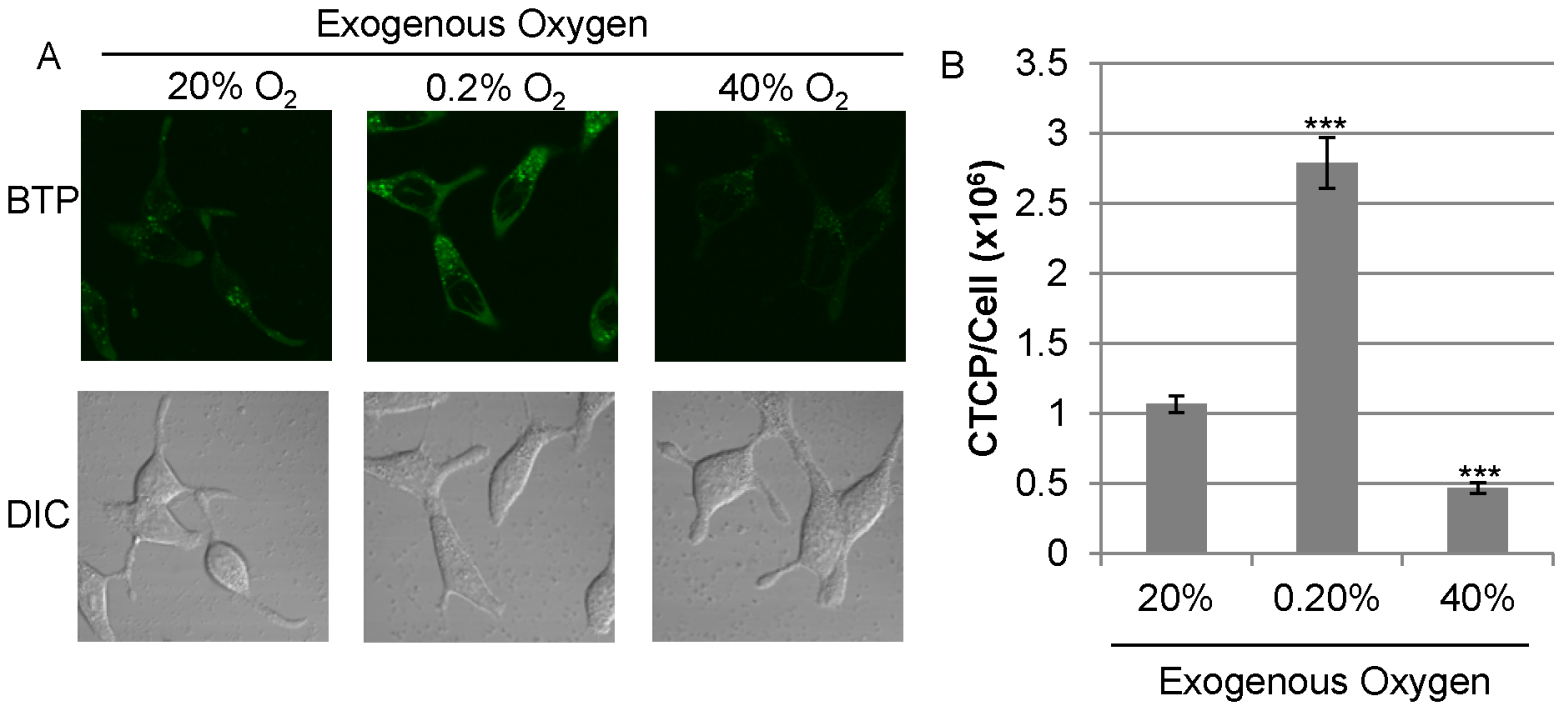
Exogenous hypoxia- and hyperoxia-induced changes in intracellular oxygen concentration. (A) BTP phosphorescence of LNCaP cells cultured at normoxia (20% O_2_), hypoxia (0.2% O_2_), or hyperoxia (40% O_2_) for 1 hour. (B) Quantification of results in panel A (*n = *10, ***P<0.001 when compared to 20% O_2_ control). Error bars represent standard error.

**Figure 7:**
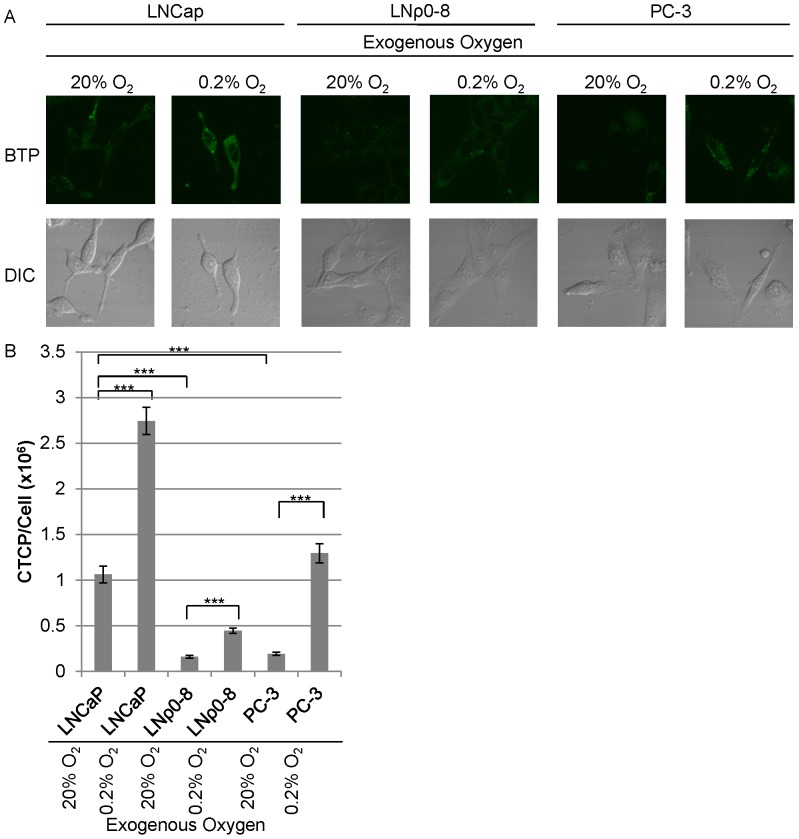
Roles of mitochondrial respiratory function on the induction of intracellular hypoxia under exogenous hypoxic condition. (A) BTP phosphorescence of LNCaP, LNρ0-8, and PC-3 under exogenous normoxia or hypoxia. (B) Quantification of results in panel A (*n* = 10, ***P<0.001 when compared to LNCaP at 20% O_2_, ***P<0.001 when LNρ0-8 at 20% O_2_ is compared to LNρ0-8 at 0.2% O_2_, ***P<0.001 when PC-3 at 20% O_2_ is compared PC-3 at O.2% O_2_). Error bars represent standard error.

We investigated how the change in exogenous oxygen concentration affects intracellular hypoxic status. First, we exposed LNCaP cells to exogenous hypoxia (0.2% O_2_) or hyperoxia (40% O_2_) for 1 hour. Incubation under normal oxygen conditions (20% O_2_) served as a control. Exposure of LNCaP cells to hypoxia significantly increased BTP phosphorescence indicating an increase in intracellular hypoxia (Fig. 6A–B, P<0.001, *n* = 10). Conversely, BTP phosphorescence was greatly reduced when LNCaP was exposed to exogenous hyperoxia (Fig. 6A–B, P<0.001, *n* = 10) indicating a shift from intracellular hypoxia to normoxia. We next examined the effects of exogenous hypoxia (0.2% O_2_) on LNCaP, PC-3, and LNρ0-8. LNCaP, PC-3, and LNρ0-8 incubated for 1 hour under normal culture conditions (20% O_2_) served as controls. LNρ0-8 and PC-3 showed limited BTP phosphorescence (Fig. 7A–B, P<0.001, *n* = 10) indicating intracellular normoxia under 20% O_2_ relative to LNCaP. We exposed LNCaP, PC-3, and LNρ0-8 to exogenous hypoxia (0.2% O_2_) for 1 hour. Exogenous hypoxia slightly increased BTP phosphorescence in LNρ0-8 but to a far less than that seen in LNCaP in the normoxic condition, suggesting that exogenous hypoxia in LNρ0-8 was not sufficient to induce strong intracellular hypoxia as observed in LNCaP even under normoxic conditions (Fig. 7A–B, P<0.001, *n = *10). PC-3 showed a great increase in BTP phosphorescence with exogenous hypoxia (Fig. 7A–B, P<0.001 when compared to normoxic PC-3, *n* = 10). BTP phosphorescence in PC-3 under the exogenous hypoxic condition is higher than that in LNCaP under normoxic condition (Fig. 7A–B). These results demonstrate the induction of strong intracellular hypoxia by exogenous hypoxia in PC-3 but not in LNρ0-8 (Fig. 7A–B). We believe that the observed differences can attributed to mitochondrial respiratory function, as PC-3 still possess a small amount of mitochondrial respiratory function (see above) but LNρ0-8 does not due to a complete absence of mtDNA [4]. We expect that the intracellular oxygen concentration in LNρ0-8 under the exogenous 0.2% oxygen concentration is the same or less than 0.2%. Since BTP phosphorescence in LNCaP under normoxic condition is higher than that in LNρ0-8 under the exogenous hypoxic condition (0.2% oxygen), intracellular oxygen concentration in LNCaP under exogenous normoxic condition should be less than 0.2% oxygen. These results demonstrate that mitochondrial respiratory function is a key regulator for the induction of intracellular hypoxia. To further explore the mechanism, we examined various nutrients in the culture media, such as, glucose and cellular growth regulators, such as, androgen for their effects on intracellular oxygen concentration.

### Glucose can Regulate Hypoxia in a Parabolic Dose-dependent Fashion

**Figure 8:**
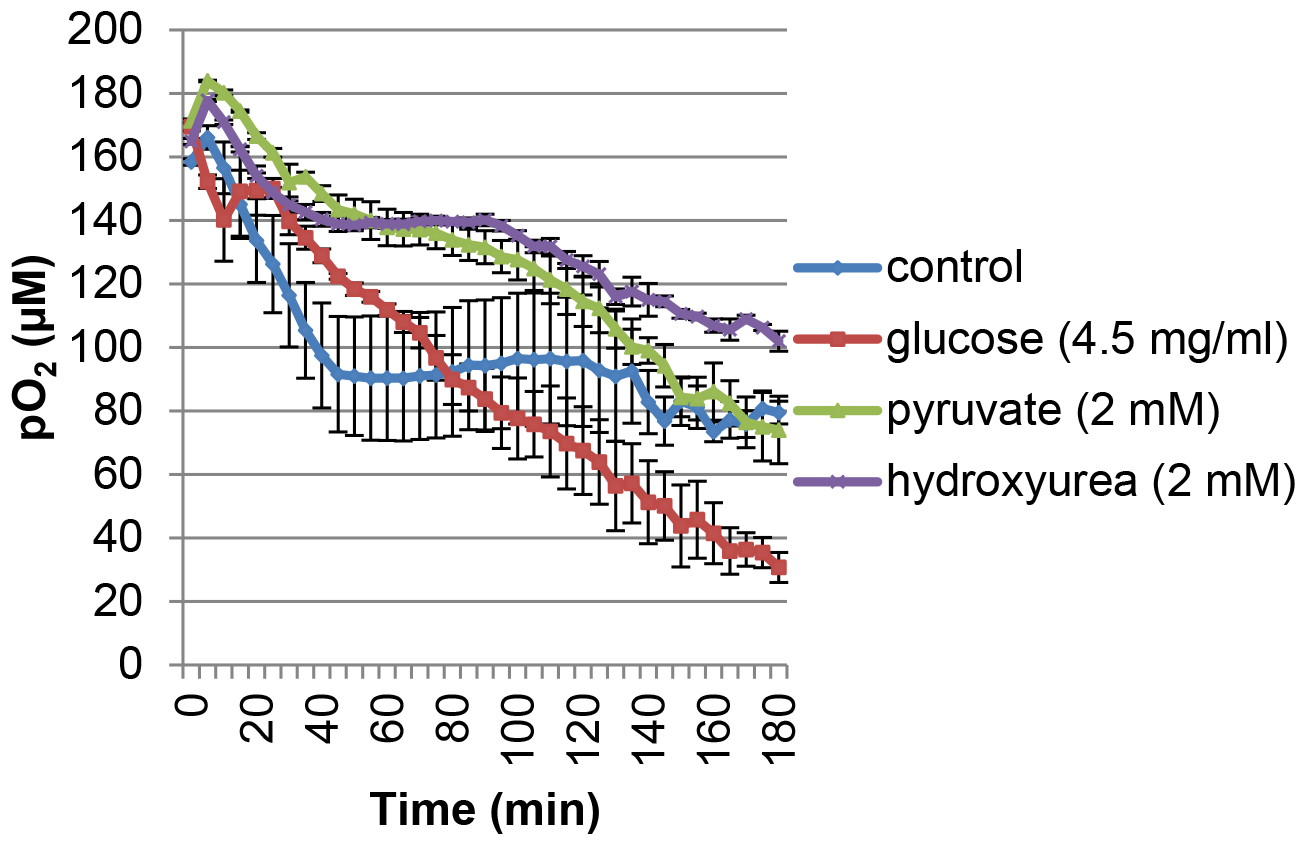
Glucose regulated oxygen concentration surrounding the cells. Cells were incubated in the presence of glucose, pyruvate, or hydroxyurea in glucose and pyruvate-free DMEM medium (this media alone served as a control). Oxygen concentration surrounding cells was measured using OxoPlate.

**Figure 9:**
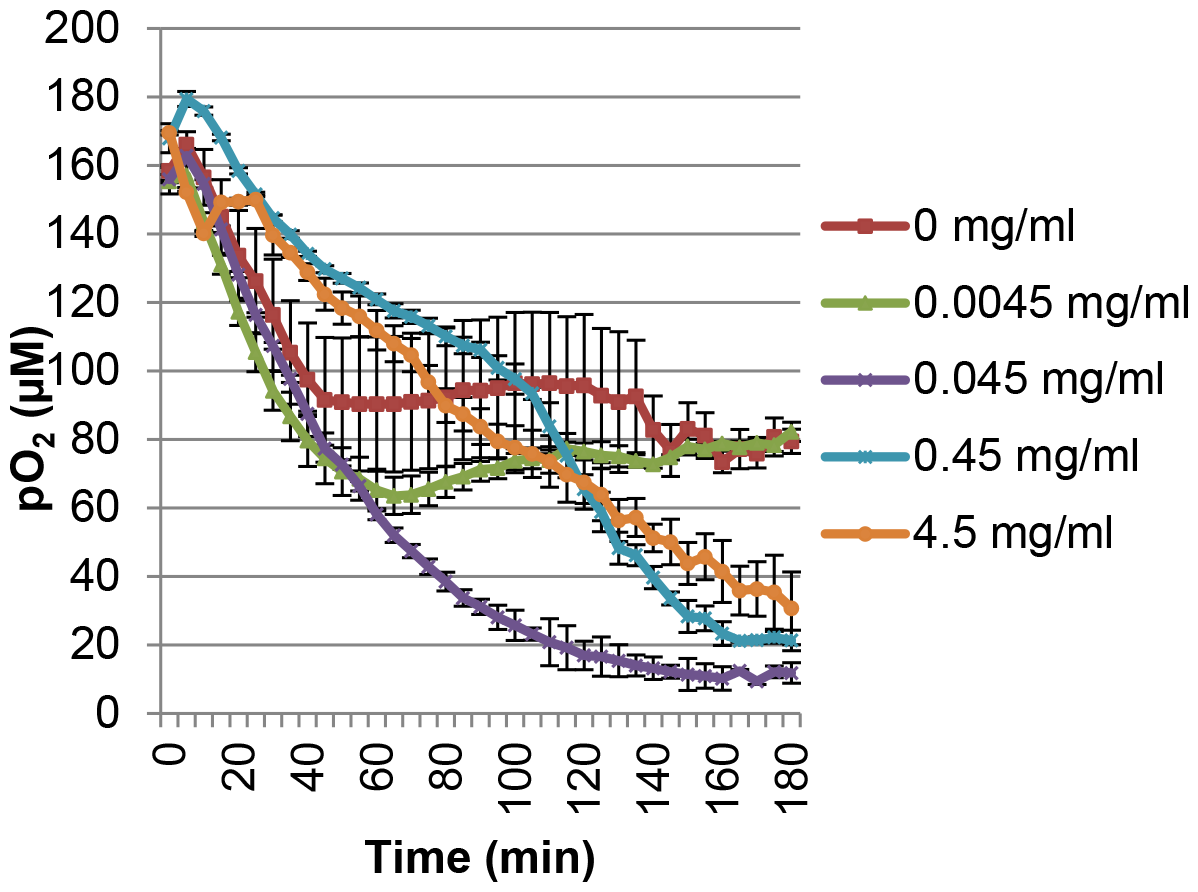
Glucose impacted hypoxia surrounding the cells in a parabolic fashion. Cells were incubated in the presence of varying concentrations of glucose. Oxygen concentrations surrounding the cells were measured using OxoPlate.

**Figure 10:**
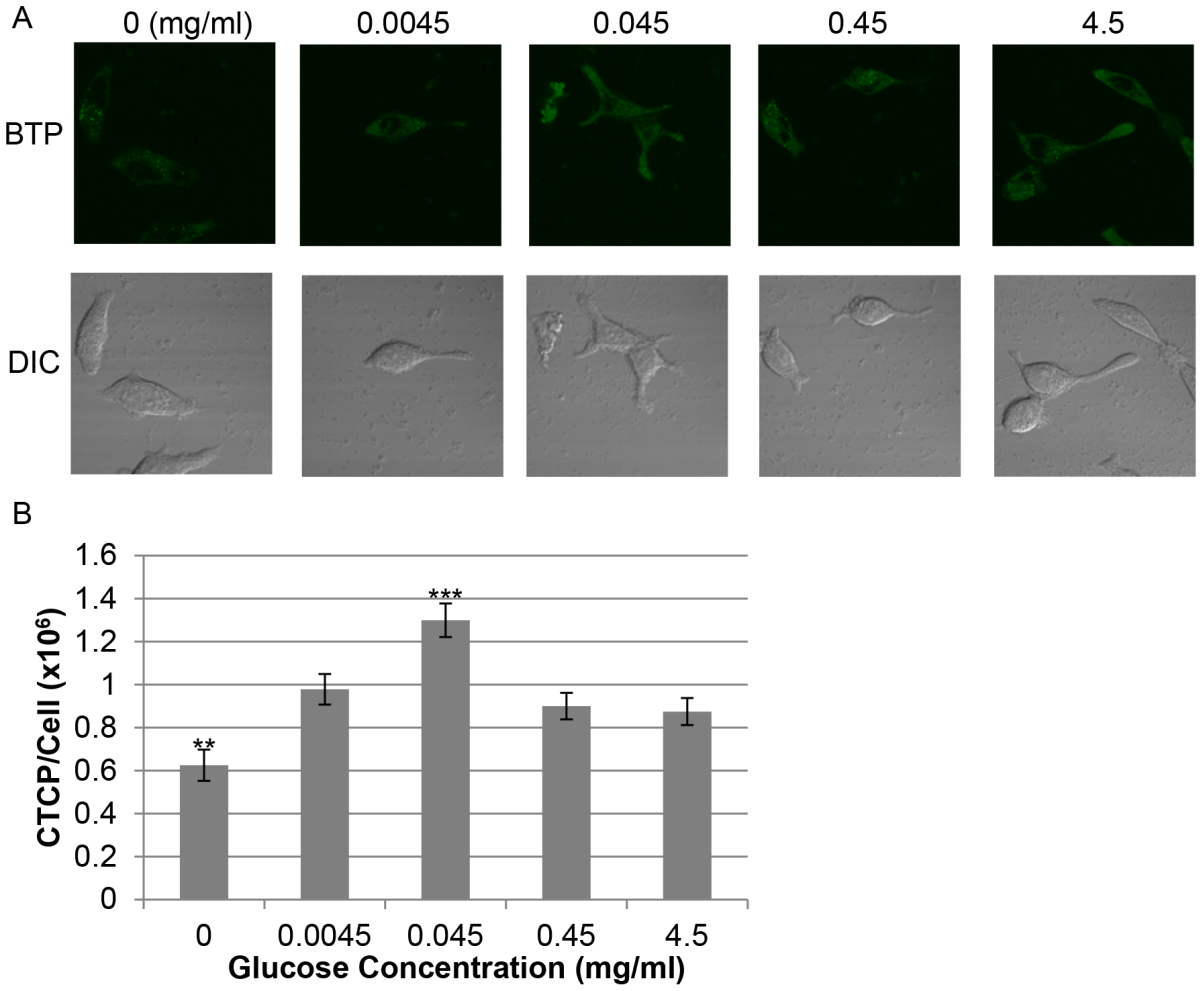
Glucose concentration regulated intracellular hypoxia in a parabolic fashion. (A) BTP phosphorescence of the cells treated with varying concentrations of glucose. (B) Quantification of results in panel A (*n* = 10, **P<0.01, ***P<0.001 when compared to 4.5 mg/ml glucose). Error bars represent standard error.

Using Oxoplate we found that 4.5 mg/ml of glucose (normal glucose concentration contained in DMEM) could induce hypoxia surrounding cells in LNCaP where other potential sources of oxidative phosphorylation (pyruvate and hydroxyurea) could not (Fig. 8). Experiments were carried out in glucose- and pyruvate-free DMEM medium plus dialyzed FCS. In Fig. 8, samples in the presence of hydroxyurea or pyruvate showed a weak decrease in oxygen concentration surrounding the cells as compared with no glucose control and with the normal glucose concentration (4.5 mg/ml). The glucose-free control showed a strong decrease in extracellular oxygen concentration as compared with samples with 4.5 mg/ml of glucose followed by a plateau at the 40 minute time point (Fig. 8). We then investigated the effect of glucose concentration on oxygen concentration surrounding cells and oxygen consumption. The ability of LNCaP to induce hypoxia surrounding the cells is greatly reduced by the complete depletion of glucose when compared to the 4.5 mg/ml control and showed a steep decrease in oxygen concentration surrounding the cells followed by a plateau at the 40 minute time point (Fig. 9). 0.45 mg/ml (10% of DMEM glucose concentration) and 0.0045 mg/ml (0.1% of DMEM glucose concentration) induced a moderately increased and decreased ability of the cells to induce hypoxia surrounding the cells, respectively, relative to control (4.5 mg/ml) (Fig. 9). Additionally, 0.0045 mg/ml of glucose showed a similar pattern to that of the zero glucose sample with a strong initial decrease in extracellular oxygen followed by a plateau at the 60 minute time point (Fig. 9). The sharp decrease in extracellular oxygen in the low or no glucose conditions may be caused by utilization of available glucose for oxidative phosphorylation to generate energy. The plateau observed in the zero and low glucose conditions may be due to the depletion of remaining glucose. At the 0.45 mg/ml concentration of glucose there was a slight increase in hypoxia surrounding the cells relative to the 4.5 mg/ml of glucose control (Fig. 9). 0.045 mg/ml glucose was found to be the strongest inducer of hypoxia surrounding the cells (Fig. 9). These data indicate that glucose dosage influences the induction of hypoxia surrounding the cells by LNCaP in a parabolic fashion (Fig. 9). Additionally, oxygen consumption rate was maximal for 0.045 mg/ml of glucose when compared with 4.5 and 0 mg/ml of glucose in agreement with the Oxoplate results (Figure S2). We then investigated the effects of glucose concentration on intracellular hypoxia as determined by BTP. 0.045 mg/ml glucose induced the strongest BTP phosphorescence indicating the induction of strongest intracellular hypoxia of the concentrations tested relative to the 4.5 mg/ml of glucose control (Fig. 10A–B, P<0.001 when compared to control, *n* = 10). 0.45 and 0.0045 mg/ml of glucose both produced BTP phosphorescence that was lower than 0.045 mg/ml of glucose indicating that the relationship between glucose availability and intracellular oxygen concentration is parabolic (a bell-shaped curve in terms of glucose concentration versus level of BTP phosphorescence). This parabolic relationship in terms of intracellular oxygen concentration and glucose availability may be due to the Crabtree effect (the inhibition of oxidative phosphorylation in high glucose concentrations [31], [32]). The cell is largely depend on glycolysis when glucose is plentiful but resorts to oxidative phosphorylation when glucose starts to become limiting. This switch to oxidative phosphorylation under low glucose conditions is advantageous to the cell since the amount of ATP produced by oxidative phosphorylation is extremely high in comparison to the ATP yield from glycolysis. At high glucose concentrations it is more advantageous to produce energy by glycolysis rather than by oxidative phosphorylation so that the cell has adequate energy production without the production of harmful reactive oxygen species in the mitochondrial respiratory chain; this is considered to be caused by the Warburg effect [33].

### Androgen is One of the Regulators of Intracellular Oxygen Concentration

**Figure 11:**
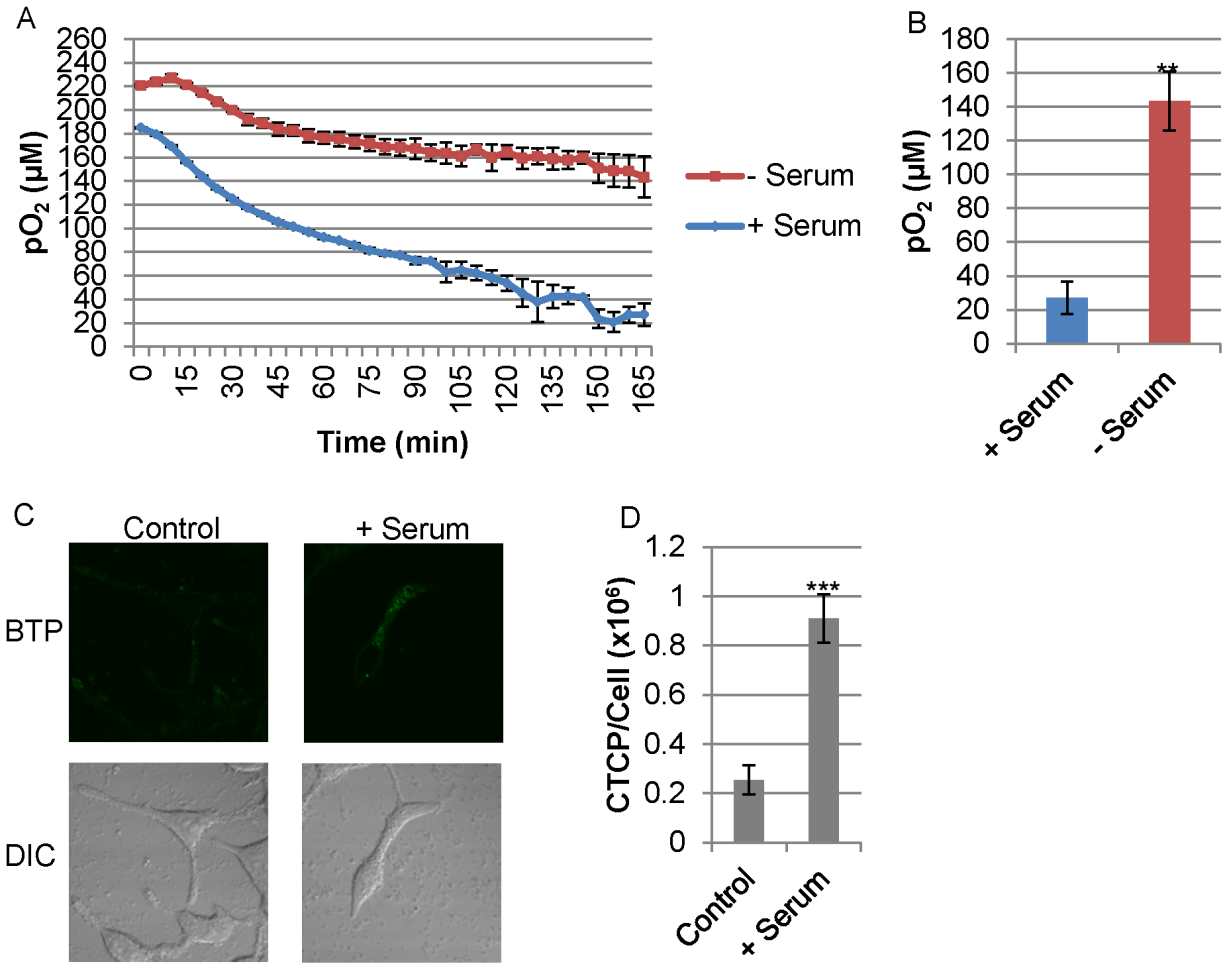
FCS induced hypoxia. (A) LNCaP cells were incubated with or without FCS. Oxygen concentration surrounding cells was measured using OxoPlate. (B) Final oxygen concentrations surrounding the cells at 180 minutes from panel A (*n* = 3, **P<0.01 when compare to minus serum). (C) BTP phosphorescence in LNCaP after incubation with or without FCS. (D) Quantification of results in panel C (*n* = 10, ***P<0.001 when compared to control). Error bars represent standard error.

**Figure 12:**
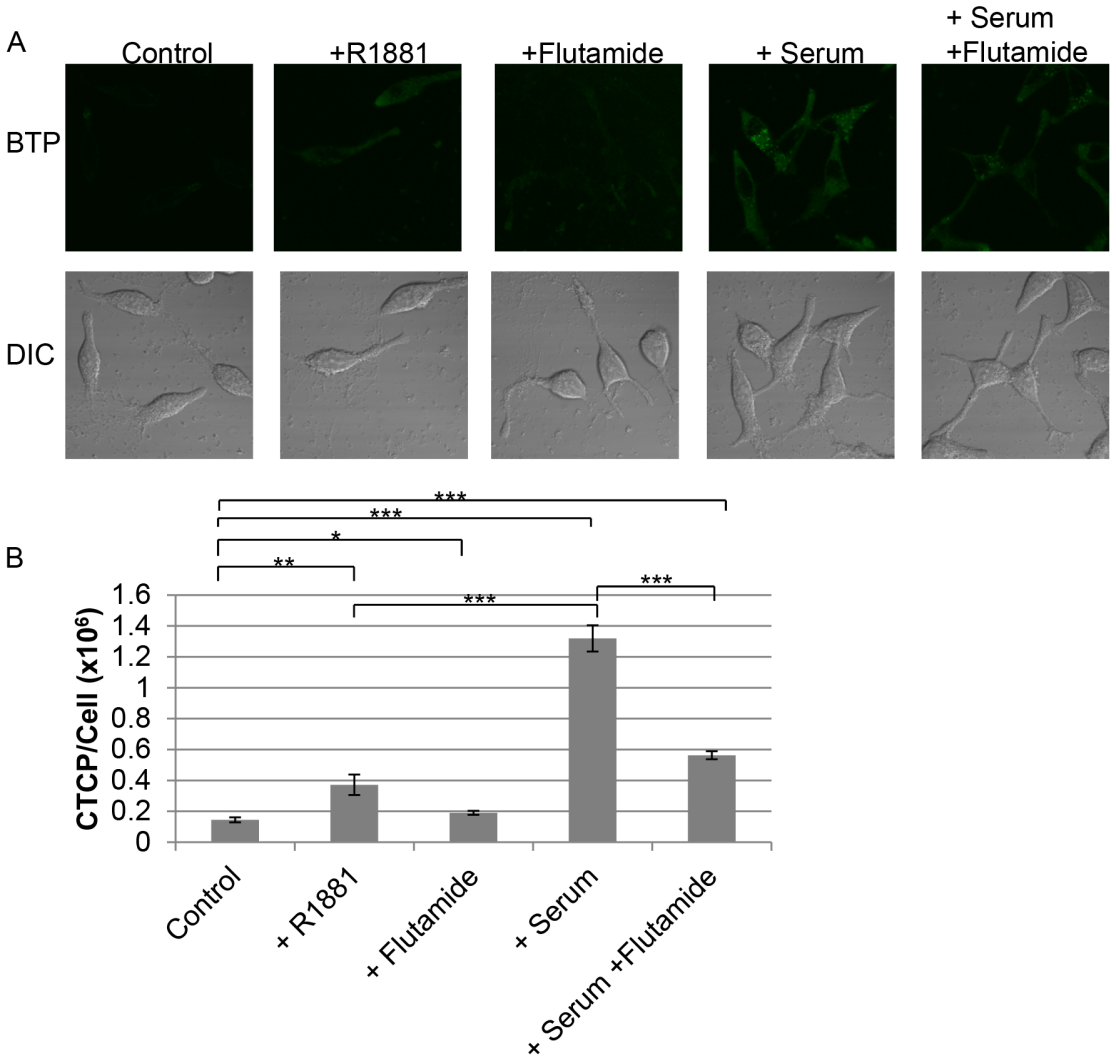
Androgen played a major role in the modulation of intracellular hypoxia in LNCaP. (A) LNCaP cells were incubated with indicated conditions and BTP phosphorescence was detected. (B) Quantification of results in panel A (*n* = 10, *P<0.05, **P<0.01, ***P<0.001 when compared to control, ***P<0.001 when+serum is compared to+R1881– serum, ***P<0.001 when+serum is compared to+flutamide+serum). Error bars represent standard error.

Estrogens have been shown to be modulators of mitochondrial respiratory activity, and androgen receptor has been shown to influence metabolism in prostate cancer [34], [35]. However, the effects of androgen on the mitochondria remain largely undocumented [25]. Both estrogen receptor and androgen receptor have been shown to localize to the mitochondria [36]–[38]. Therefore, we hypothesized that androgen may be a modulator of mitochondrial function in LNCaP cells. As shown in Fig. 11A–B, LNCaP in the presence of FCS showed a stronger ability to induce hypoxia surrounding the cells than the FCS-free control (P<0.01, *n* = 3). In agreement with this finding, LNCaP in the presence of FCS showed stronger BTP phosphorescence than that in the absence of FCS, indicating stronger intracellular hypoxia in the presence of FCS (Fig. 11C–D, P<0.001, *n* = 10, when compared to the FCS-free control). The association of estrogen, androgen, and estrogen receptor on mitochondrial function have been implicated [39], and LNCaP has been demonstrated to be androgen sensitive [19]. However, whether estrogen and androgen can affect intracellular hypoxic status have yet to be demonstrated. Therefore, we hypothesized that androgen affects intracellular hypoxia in LNCaP cells. In microscopic studies using BTP, R1881, a synthetic androgen, increased phosphorescence to approximately four times the control level indicating that R1881 induced hypoxia in the absence of FCS (Fig. 12A–B, P<0.01, *n* = 10). Addition of flutamide, an inhibitor of the androgen-androgen receptor binding, in the presence of FCS reduced BTP phosphorescence indicating that flutamide reduced intracellular hypoxia to a level similar to the FCS-free control, possibly by blocking small amounts of androgen present in the serum or synthesized by LNCaP (Fig. 12A–B, P<0.001, *n* = 10) [40], [41]. The normal concentration of total testosterone (androgen) in RPMI supplemented with 10% normal FCS has been reported to range from 55.1 to 97.5 pM; normal human males have a total testosterone level of 10–35 nM [42]. Flutamide in the absence of serum showed a slight increase in BTP phosphorescence relative to the serum-free control (CTCP/Cell: 190200±13038 (SE) % versus 144549±16316 (SE) %, respectively) (Fig. 12A–B, P<0.05 when compared to serum-free control, *n* = 10) implicating androgen synthesis from LNCaP. These data are the first to indicate that androgen is one of the modulators of cellular respiration and intracellular oxygen level.

Here it should be pointed out that *in vivo* oxygen concentration within tissues ranges from 0% to 14%, depending on the tissue, indicating the potential roles of exogenous hypoxia in inducing intracellular hypoxia [43]. We showed that LNCaP induces strong endogenous intracellular hypoxia even under normal culture conditions (20% O_2_, 5% CO_2_). PC-3 induces strong hypoxia only under exogenous hypoxia. However, cells with no mtDNA failed to induce strong intracellular hypoxia even under exogenous hypoxia. These findings implicate that oxygen consumption via mitochondrial respiration is required to induce strong intracellular hypoxia under exogenous hypoxic condition. Our results also suggest that endogenous intracellular hypoxia-induced HIF-1α activation (Fig. 5C–D) is dependent on mitochondrial respiratory function and may regulate many hypoxia-related cell processes. Since the K_m_ of cytochrome a+a3, the center for consuming oxygen in mitochondrial respiratory chain complex IV, is very low and the reaction is very rapid [44], reduction of the intracellular oxygen concentration by the consumption of oxygen by cytochrome a+a3 can likely inhibit enzymatic activity of other oxygen requiring enzymes such as P450 and Cyp51 [5], [44], [45] by reducing oxygen availability. Additionally recent findings show that the protein synthesis machinery is also regulated by intracellular oxygen concentration [46]. The fact that androgen can regulate intracellular oxygen concentration indicates that androgen can regulate oxygen requiring enzymes. Glucose and androgen may be working synergistically to increase oxygen consumption.

An interesting trend was noticed in our data. Well differentiated cell lines such as LNCaP and MCF-7 [19], [23] showed strong hypoxia-inducing ability. The moderately differentiated cell line, C4-2 [22], showed a moderate ability to induce extracellular and intracellular hypoxia. The poorly differentiated cell lines, PC-3 [21] and MDAMB231 [24], had only a limited ability to induce extracellular and intracellular hypoxia. This trend in *in vitro* cell lines suggests that, at least in prostate cancer, and possibly in breast cancer, the degree of cancer progression can be related to cellular oxygen status. In combination with our previous findings linking decreased oxygen consumption in prostate cancer leading to the activation of Ras [4], our findings open up new avenues of investigation of the pathophysiology and the progression of prostate cancer.

## Supporting Information

Figure S1 **Diffusion of oxygen into water containing 2.5 mg/ml of sodium sulfite with or without layered mineral oil.**
(TIF)

Figure S2 **Oxygen consumption of LNCaP cells under different glucose conditions.** Maximal oxygen consumption rates of LNCaP cells as measured using Oxytherm under different concetnrations of glucose in the presences of dialyzed FCS.
(TIF)

File S1 **Materials and methods for Figures S1 and S2 are detail in File S1.**
(DOCX)
