# Supplementary figures and images for: Mitochondrial Respiratory Function Induces Endogenous Hypoxia
Source: PLoS One. 2014 Feb 21;9(2):e88911. doi: 10.1371/journal.pone.0088911 (PMC3931703; doi:10.1371/journal.pone.0088911)

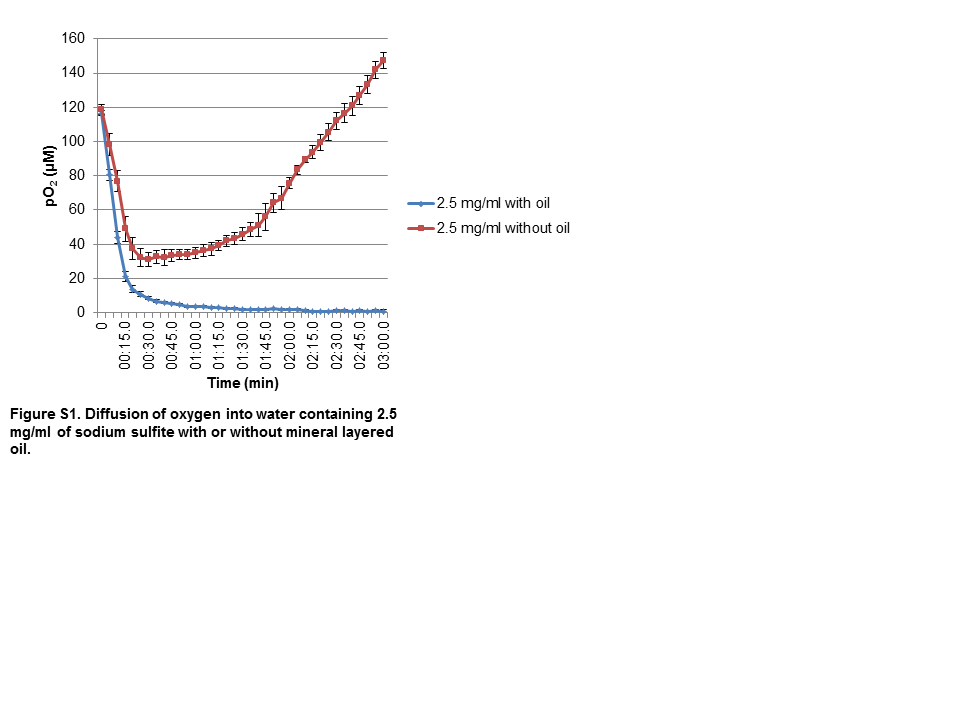

Supplement: Figure S1 — Diffusion of oxygen into water containing 2.5 mg/ml of sodium sulfite with or without layered mineral oil. (TIF) [file pone.0088911.s001.tif]
