# Supplementary material for: Mitochondrial Respiratory Function Induces Endogenous Hypoxia
Source: PLoS One. 2014 Feb 21;9(2):e88911. doi: 10.1371/journal.pone.0088911 (PMC3931703; doi:10.1371/journal.pone.0088911)
Supplement: File S1 — Materials and methods for Figures S1 and S2 are detail in File S1. (DOCX) [file pone.0088911.s003.docx]

**S3. Experimental Procedures**

*Oxygen concentration in 2.5 mg/ml sodium sulfite solution with or without layered mineral oil.* The Oxoplate system is described in detail in the Experimental Procedures section. Briefly wells were filled with 200 µl of water containing 2.5 mg/ml of sodium sulfite. Three wells were left open to the environment and three were covered with 50µl of mineral oil to prevent majority of the diffusion of oxygen in to the fraction of sodium sulfite solution. Oxygen concentration at the bottom of the wells was read every 5 minutes for 3 hours as described, and control saturated- and depleted-oxygen wells were setup as described in the Experimental Procedures (*n* = 3).

*Oxygen Consumption under varying glucose conditions.* LNCaP cells were cultured under normal incubation conditions in 10 cm dishes. Cells were trypsinized and washed twice with PBS to remove residual traces of normal serum and glucose. 2x10^6^ cells were resuspended in 500µl glucose-free DMEM plus 4.5, 0.045, or 0 mg/ml glucose and 5% dialyzed FCS. Samples were kept at 4 ^O^C to keep cell metabolism to a minimum until the samples could be analyzed. Before the cells were added to the respiration chamber, 500 µl of pre-warmed media with the appropriate glucose concentration and 5% dialyzed FCS was added to each sample. Oxygen consumption rate was then measured using an oxygen electrode as described in the Experimental Procedures. Oxygen levels in the chamber were measured until they reached 0 pM, and maximal oxygen consumption rate was then determined for each sample (*n* = 1).
